# Unveiling the Roles of LncRNA *MOIRAs* in Rice Blast Disease Resistance

**DOI:** 10.3390/genes15010082

**Published:** 2024-01-09

**Authors:** Qing Liu, Jiao Xue, Lanlan Zhang, Liqun Jiang, Chen Li

**Affiliations:** 1 Guangdong Key Laboratory of New Technology in Rice Breeding, Guangdong Rice Engineering Laboratory, Key Laboratory of Genetics and Breeding of High Quality Rice in Southern China (Co-Construction by Ministry and Province), Ministry of Agriculture and Rural Affairs, Rice Research Institute, Guangdong Academy of Agricultural Sciences, Guangzhou 510640, China; 15033254071@163.com (L.Z.); jiangliqun@gdaas.cn (L.J.); lichen@gdaas.cn (C.L.); 2 Guangdong Key Laboratory for Crop Germplasm Resources Preservation and Utilization, Agro-Biological Gene Research Center, Guangdong Academy of Agricultural Sciences, Guangzhou 510640, China; xuejiao@gdaas.cn

**Keywords:** rice blast disease, lncRNAs, *Magnaporthe oryzae*, disease resistance, crop yield

## Abstract

Rice blast disease, caused by the fungal pathogen *Magnaporthe oryzae*, is a major threat to rice production worldwide. This study investigates the role of long non-coding RNAs (lncRNAs) in rice’s response to this destructive disease, with a focus on their impacts on disease resistance and yield traits. Three specific lncRNAs coded by *M. oryzae* infection-responsive lncRNAs (*MOIRAs*), *MOIRA1*, *MOIRA2*, and *MOIRA3*, were identified as key regulators of rice’s response to *M. oryzae* infection. Strikingly, when *MOIRA1* and *MOIRA2* were overexpressed, they exhibited a dual function: they increased rice’s susceptibility to blast fungus, indicating a negative role in disease resistance, while simultaneously enhancing tiller numbers and single-plant yield, with no adverse effects on other yield-related traits. This unexpected improvement in productivity suggests the possibility of overcoming the traditional trade-off between disease resistance and crop yield. These findings provide a novel perspective on crop enhancement, offering a promising solution to global food security challenges by developing rice varieties that effectively balance disease resistance and increased productivity.

## 1. Introduction

Plant diseases pose a significant and widespread threat to global agriculture, presenting the potential to devastate crop yields and, consequently, exert far-reaching economic consequences. The adaptability of plants to a multitude of pathogens and pests is a pivotal factor in the preservation of crop viability and productivity. The complex and dynamic relationships between plants and pathogens have engendered an ongoing evolutionary battle, as plants continuously evolve novel defense mechanisms with which to combat encroaching threats. In this context, unraveling the intricate molecular foundations of these interactions is of utmost significance. The relationships between plants and pathogens can be likened to an evolutionary arms race. Pathogens continually devise new strategies to infiltrate and infect plants, while plants, in turn, develop innovative ways to resist and counter these attacks. This ongoing battle has driven the co-evolution of both plants and pathogens, resulting in a dynamic and ever-changing biological landscape [1,2]. Understanding the underlying mechanisms of these diseases and their interactions with crops is essential for safeguarding our global food supply.

Rice, one of the world’s most vital staple crops, is highly susceptible to numerous diseases, with rice blast disease, caused by the fungal pathogen *M. oryzae*, standing out as one of the most devastating. Termed the “cancer of rice”, this disease has the capacity to devastate entire rice crops, impacting grain yield, the economy, and global food security [3]. The extensive implications of rice blast disease have made it a focal point for life science and agricultural research. Rice blast disease’s devastating impact is primarily due to its ability to infect various parts of the rice plant, including the leaves, stems, and panicles. This pathogen, *M. oryzae*, employs various strategies through which to infiltrate and colonize rice plants, adapting to changing conditions and developing new strains, making it a persistent and evolving threat [4]. Given its profound impact on rice, rice blast disease has assumed a central position in scientific and agricultural research. Developing effective strategies to control this disease is not only vital for crop protection, but also has broader implications for food production and agricultural sustainability.

Historically, research efforts aimed at understanding and combating rice blast disease have predominantly centered on protein-coding genes. These genes have been meticulously studied for their roles in plant defense mechanisms, but the ever-evolving dynamics between plants and pathogens suggest that other, less-explored components of the genome may also be pivotal in these interactions. One such component is non-coding RNA. Non-coding RNAs (ncRNAs) constitute a substantial portion of the eukaryotic transcriptome, and their functions are becoming increasingly appreciated in various biological processes. Importantly, ncRNAs, and particularly long non-coding RNAs (lncRNAs), have been recognized as critical regulators of the plant’s defense mechanisms against diseases and pests [5,6,7,8]. Recent research has unveiled their capacities to modulate gene expression, shape chromatin structure, and influence cellular processes in response to pathogen invasion [9,10,11,12,13]. A report indicates that, in Arabidopsis, an lncRNA acts as a crucial regulator, striking a balance between immunity and growth. This lncRNA fine-tunes the production of salicylic acid (SA) by controlling the expression of a nearby transcription factor responsible for SA biosynthesis. When the plant is infected by a pathogen, the lncRNA’s expression decreases, leading to the release of immune responses [14]. In another case, an lncRNA called *Sl-lncRNA15492* interacts with Sl-miR482a, influencing the immune response of *Solanum lycopersicum* (tomato) against *Phytophthora infestans* [15]. Additionally, tomato *lncRNA23468* serves as a competing endogenous RNA, manipulating NBS-LRR genes by drawing miR482b away in the tomato–*P. infestans* interaction [16]. In cotton plants, *lncRNA7* and *lncRNA2* are involved in adjusting cell wall defense genes to regulate resistance against Verticillium wilt. Furthermore, the induction of *MSTRG18363* expression in tomato plants by SL18r promotes the decoy of miR1918 and regulates the expression of *SlATL20*, thereby initiating ISR responses against foliar pathogen infections [17]. In the cases of two central lncRNAs, *GhlncNAT-ANX2*- and *GhlncNAT-RLP7*-silenced seedlings exhibit increased resistance to *Verticillium dahliae* and *Botrytis cinerea*, likely due to the enhanced expressions of *LOX1* and *LOX2* [18].

Presently, several studies have delved into the realm of lncRNAs in the context of rice blast disease. In one investigation, a total of 161 rice blast fungus-responsive lncRNAs in rice were unveiled, offering insights into their potential roles in orchestrating the interactions between rice and the blast fungus, particularly within the framework of JA-mediated rice resistance to the pathogen [19]. Another research effort introduced a computational pipeline for the identification of lncRNAs in a resistant rice line, revealing 1429, 1927, and 1981 lncRNAs in mock and *M. oryzae* (ZB13 and Zhong)-inoculated samples, respectively [20]. Moreover, the exploration into infection-associated lncRNAs employed lncRNA profiling across six stages of host infection, spanning from vegetative growth and pre-penetration to the biotrophic and necrotrophic stages, in *M. oryzae*. In this endeavor, 2601 novel lncRNAs were brought to light, encompassing 1286 antisense lncRNAs and 980 intergenic lncRNAs. Among these newly identified lncRNAs, 755 displayed stage-specific expression, with an additional 560 being uniquely expressed during infection [21]. Nevertheless, a conspicuous gap in our knowledge remains, regarding the precise functions of these lncRNAs in aiding rice’s defense against the disease.

In this study, we embark on an exploration of the uncharted territory of lncRNAs in the context of rice blast disease. Specifically, we identified and characterized three lncRNAs that respond to the infection of *M. oryzae*. We christened these lncRNAs as *M. oryzae* infection-responsive lncRNAs, abbreviated as *MOIRA1*, *MOIRA2*, and *MOIRA3*. Through a series of experiments and analyses, we unraveled their role in rice’s defense mechanisms and unveiled their influence on disease resistance and yield. Our findings not only shed light on the molecular mechanisms of the rice–*Magnaporthe* interaction, but also have important implications for crop improvement and food security.

## 2. Materials and Methods

### 2.1. Plant Materials, Growth Conditions, and Pathogen

The wild-type rice cultivar Zhonghua 11 (ZH11, ssp. *japonica*) served as the basis for generating the overexpressing transgenic plants in this study. All of the wild-type and overexpressing plants were carefully cultivated within the controlled environment of a greenhouse, which was maintained at a temperature of 28 °C, and adhered to a 12 h light/12 h dark cycle with a light intensity of 200 μmol photons m^−2^ s^−1^. The *M. oryzae* Guy11 strain, which was sourced from South China Agricultural University, was cultured on prune agar medium at a temperature of 28 °C for a duration of 3 days under dark conditions [22]. Subsequently, the strain was subjected to a 12 h light/12 h dark photoperiod for 4 days to stimulate conidiation.

### 2.2. Pathogen Inoculation

To initiate pathogen inoculation, the first leaves of both the wild-type and overexpressing transgenic plants, at the four-leaf stage, were carefully excised and placed within square petri dishes. To maintain adequate humidity, two strips of defatted cotton, soaked in sterile ultra-pure water containing 2 μg·mL^−1^ kinetin (KT, Promega, Shanghai, China), were positioned at both ends of the leaves. Subsequently, three petri dishes containing the fungus were selected and inverted (with the mycelium side facing down) onto one leaf for the inoculation process. Leaves that were subjected to sterile prune agar medium served as the non-inoculation (NI) control. The sealed petri dishes, covered with plastic wrap, were initially kept in darkness at a temperature of 28 °C for 24 h. Following this period, the inoculation process was carried out under a photoperiod of 16 h of light and 8 h of darkness, a condition that was maintained for a duration of 5 days. Lesion size was quantified using the ImageJ program, and the leaves subjected to inoculation, with or without the presence of fungus dishes, were carefully fixed onto white paper, using double-sided tape, for photographic documentation. Leaf samples for gene expression analysis were collected at intervals of 0, 1, 2, 3, 4, and 5 days.

### 2.3. Generation of Transgenic Plants

To generate overexpression vectors for *MOIRA1* (*XLOC_083948*), *MOIRA2* (*XLOC_419750*), and *MOIRA3* (*XLOC_428992*), the sequences of these genes were first amplified from ZH11 cDNA using specific primers, XLOC_083948-OE-F/R, XLOC_419750-OE-F/R, and XLOC_428992-OE-F/R (as listed in Supplemental Table S1). The resulting sequences were then sub-cloned into the binary vector pOx, a method previously described by Liu et al. [23]. Subsequently, these constructs were introduced into the ZH11 rice plants via Agrobacterium-mediated transformation, facilitated by *Agrobacterium tumefaciens* EHA105, with the transformation process being conducted by Wuhan Biorun Biosciences Co., Ltd. (Wuhan, China).

### 2.4. RNA Isolation and Quantitative qRT-PCR

Total RNA was extracted from the rice leaves of both wild-type and transgenic plants, with or without pathogen inoculation, using the Hipure plant RNA Mini Kit (Magen, Guangzhou, China). Subsequently, first-strand cDNA was synthesized from 1000 ng of total RNA, utilizing the PrimerScript™ RT Reagent Kit (Takara Bio Inc., Japan). The synthesized cDNA was then subjected to quantitative real-time PCR (qRT-PCR), using the SYBR Premix Extaq™ kit (Takara, Japan), following the manufacturer’s instructions. In this analysis, the rice EF1α gene served as the internal control to ensure accurate normalization of expression levels. The relative expression levels of the genes were calculated using the 2^−△△CT^ method, thus providing a reliable assessment of their responses to different experimental conditions. Detailed information on the gene-specific primers used in these experiments can be found in Supplemental Table S1.

### 2.5. Statistical Analysis

In our study, we ensured robustness and reliability by conducting three independent experiments for gene expression analysis and disease resistance evaluation. Within each experiment, we performed three biological replicates, guaranteeing the consistency and accuracy of our findings. All data presented in this study are expressed as means ± standard deviation (SD), as derived from these three biological replicates. To assess the statistical significance of our results, we employed “Dunnett’s test”, and the analysis was conducted using IBM SPSS statistics software (version 23). This rigorous statistical approach allowed us to draw meaningful conclusions from our research outcomes.

## 3. Results

### 3.1. Identification of Three LncRNAs Responsive to M. oryzae Infection

It has been previously reported that 48,727 lncRNA transcripts were identified in *Xanthomonas oryzae* pv. *oryzae* (*Xoo*)-infected rice leaves, and a subset of them has been demonstrated to be associated with the jasmonate pathway [24]. Given the well-documented influence of jasmonic acid and its derivatives on rice’s resistance to bacterial and fungal pathogens, we were eager to investigate whether lncRNAs could also play a role in the resistance to rice blast disease.

**Figure 1:**
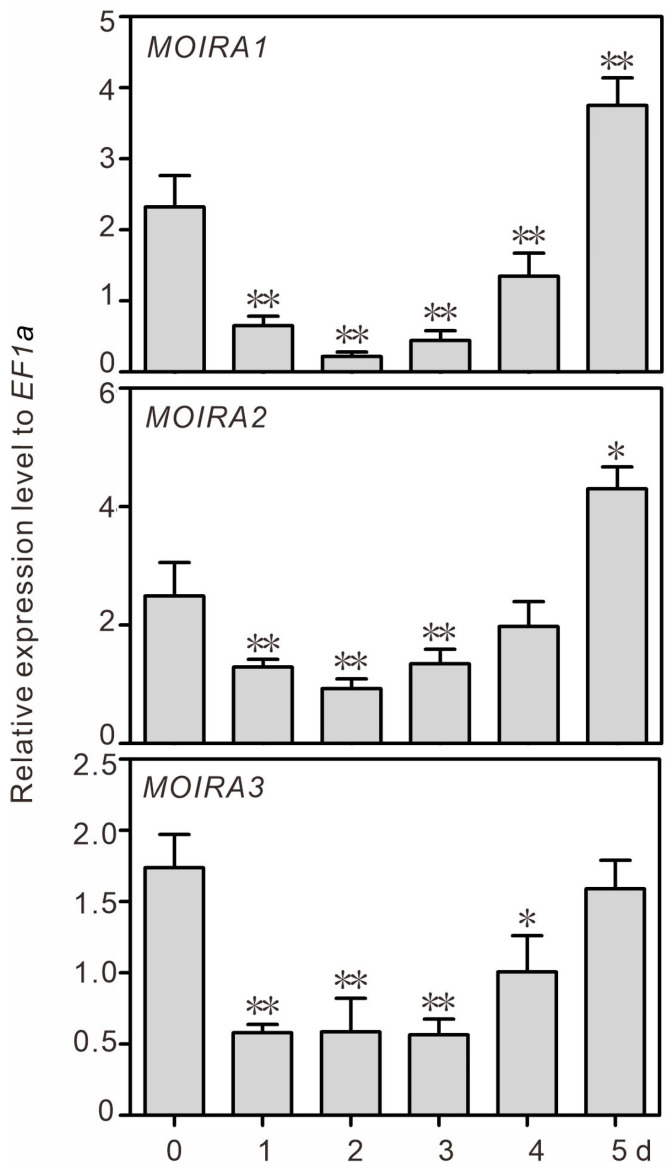
The responses of *MOIRA1*, *MOIRA2*, and *MOIRA3* to leaf blast inoculation in rice. The values represent the means ± SD of three biological replicates. The asterisks indicate significant differences compared with the 0 d time point (Dunnett’s test, ** *p* < 0.01 and * *p* < 0.05).

In the course of our investigation, we successfully identified three lncRNAs, *XLOC_083948*, *XLOC_419751*, and *XLOC_428992*, that exhibited a robust response to infection by the fungal pathogen *M. oryzae*. Our findings revealed that all three of these lncRNAs displayed a distinctive expression pattern in response to *M. oryzae* infection. Specifically, their expression levels initially decreased (during the first two days post-infection) and subsequently increased (from the third to the fifth day post-infection) (Figure 1). Notably, the expression levels of *XLOC_083948* and *XLOC_419751*, two of these lncRNAs, even exceeded their baseline levels by the fifth day. These results underscore the significant responsiveness of these three lncRNAs to *M. oryzae*, leading us to categorize them as *M. oryzae infection-responsive lncRNAs* (*MOIRAs*). Accordingly, the lncRNAs *XLOC_083948*, *XLOC_419751*, and *XLOC_428992* are now denoted as *MOIRA1*, *MOIRA2*, and *MOIRA3*, respectively, in the subsequent analyses and discussions. *MOIRA1* is situated on the antisense strand of rice chromosome 10, spanning the coordinates 19,933,213–19,933,791, and comprising two exons. *MOIRA2* is located on the sense strand of rice chromosome 8, spanning the coordinates 24,315,675–24,316,231, and transcribing a single-exon lncRNA. *MOIRA3* is positioned on the antisense strand of rice chromosome 8, with coordinates ranging from 10,531,583 to 10,531,985, and comprising two exons. The complete sequences of these three lncRNAs can be found in Supplementary Table S2.

### 3.2. Tissue-Specific Expressions of MOIRAs

**Figure 2:**
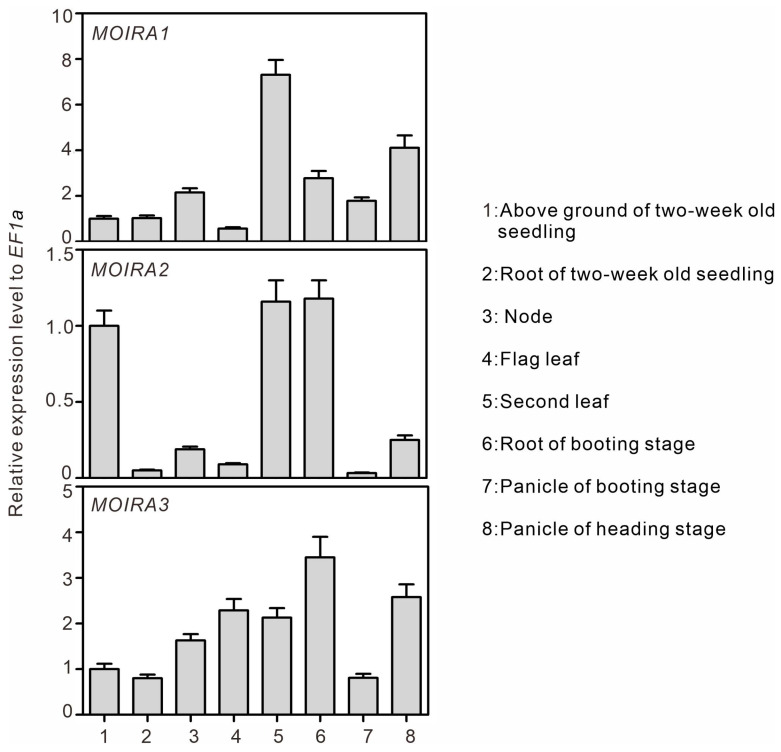
Expression patterns of *MOIRA1*, *MOIRA2*, and *MOIRA3* in different rice tissues. The values represent the means ± SD of three biological replicates.

Our investigation into *MOIRAs’* expression patterns across different rice tissues sheds light on their potential functional roles. Rice blast disease can manifest in various stages and parts of the plant, including the seedlings, leaves, nodes, leaf sheaths, necks of the panicles, and grains. Among these, leaf blast is the most prevalent form of the disease. To gain insights into *MOIRAs’* tissue-specific expressions, we collected samples from eight distinct rice tissues, including the above-ground parts of two-week-old seedlings, the root systems of two-week-old seedlings, the nodes, the flag leaves, the second-to-last leaves, the roots at the booting stage, the panicles at the booting stage, and the panicles at the heading stage. For each of the three lncRNAs—*MOIRA1*, *MOIRA2*, and *MOIRA3*—we conducted tissue expression profiling. Our findings unveiled tissue-specific expression patterns for *MOIRAs* (Figure 2). *MOIRA1* displayed higher expression levels in the second-to-last leaves, the panicles at the booting stage, and the roots at the booting stage. *MOIRA2* exhibited a similar expression pattern to *MOIRA1*, with notable expression in the second-to-last leaves, the roots at the booting stage, and the above-ground parts of two-week-old seedlings. In contrast, *MOIRA3* displayed relatively stable expression across all tissues, with its highest expression detected in the roots at the booting stage.

The observed high expressions of *MOIRAs* in various tissues, particularly in the second-to-last leaves and the roots at the booting stage, underscores their potential involvement in different aspects of rice development and defense. These expression patterns suggest that *MOIRAs* might play crucial roles in the early defense response of rice against *M. oryzae*, particularly in the aerial parts of the plant, which are often the primary targets of pathogen attacks.

### 3.3. Overexpression of MOIRAs Enhances Rice Susceptibility to Blast Fungus

**Figure 3:**
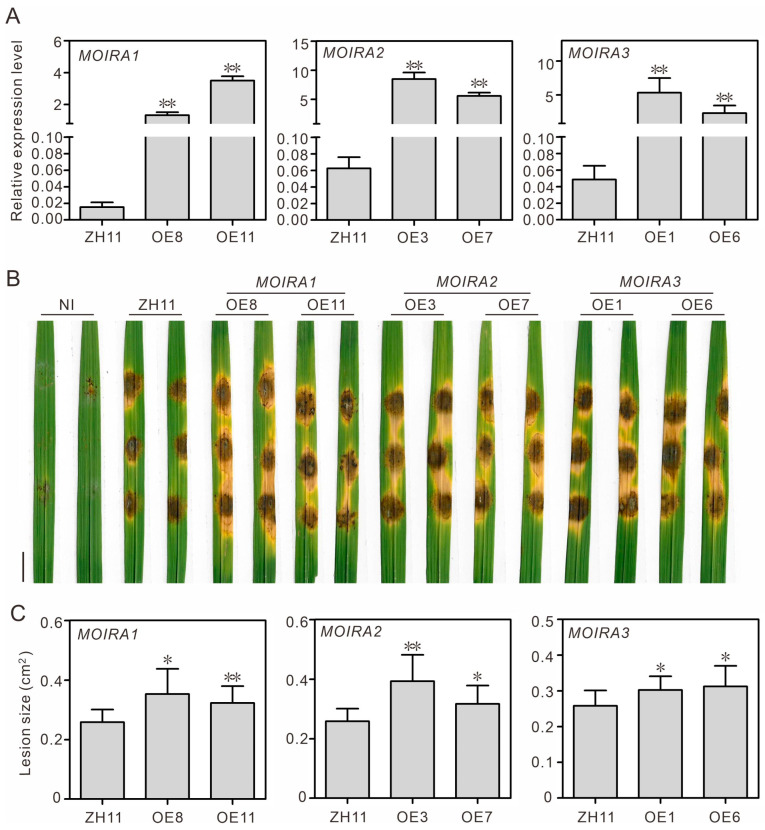
Overexpression of *MOIRA1*, *MOIRA2*, and *MOIRA3* showed decreased leaf blast resistance in rice. (**A**) Relative expression levels of *MOIRA1*, *MOIRA2*, and *MOIRA3* in the wild-type Zhonghua 11 (ZH11), and their corresponding overexpressing (OE) plants. The values represent the means ± SD of three biological replicates. The asterisks indicate significant differences compared with the ZH11 plants (Dunnett’s test, ** *p* < 0.01). (**B**) Phenotypes of the ZH11 and *MOIRA*-overexpressing plants after leaf blast inoculation for 5 d. NI indicates non-inoculation. Scale bar = 1.0 cm. (**C**) Lesion sizes of ZH11 and *MOIRA*-overexpressing plants after leaf blast inoculation for 5 d. Values are expressed as the means ± SD of three biological replicates (10 plants for each replicate), and asterisks indicate significant differences between ZH11 and overexpressing plants (Dunnett’s test, ** *p* < 0.01 and * *p* < 0.05).

**Figure 4:**
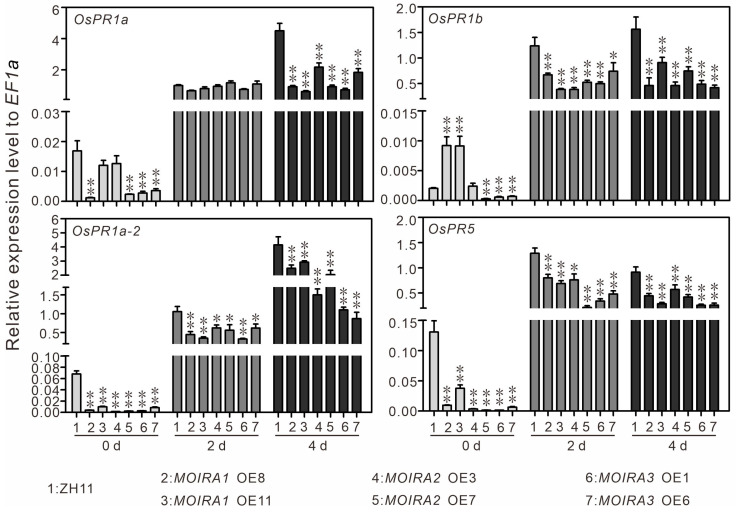
The expression patterns of four pathogen-responsive genes in the ZH11 and lncRNA-overexpressing plants before and after leaf blast inoculation. Values are expressed as the means ± SD of three biological replicates, and asterisks indicate significant differences between ZH11 and overexpressing plants (Dunnett’s test, * *p* < 0.05 and ** *p* < 0.01).

To explore the functional significance of *MOIRAs*, we conducted overexpression experiments in rice plants. Utilizing Agrobacterium-mediated rice genetic transformation, we successfully introduced *MOIRA1*, *MOIRA2*, and *MOIRA3* into rice, generating stable overexpressing plants. Subsequent qRT-PCR experiments confirmed that these three lncRNAs exhibited several hundred-fold upregulations in the overexpressing plants (Figure 3A). Following successful overexpression, we subjected these plants to blast fungus infection assays. The results were remarkable: overexpressing *MOIRAs* significantly heightened the plant’s susceptibility to blast fungus, intensifying the disease’s impact on rice (Figure 3B,C). Furthermore, we evaluated the expressions of four pathogenesis-related genes in these overexpressing plants. We found that, in the quiescent state, the majority of the overexpressed materials displayed noticeable suppression of pathogenesis-related genes (Figure 4). However, upon infection by blast fungus, the expressions of these pathogenesis-related genes in these materials increased, albeit remaining relatively lower compared to their expressions in wild-type plants. This gene expression pattern aligns with the observed phenotypic outcomes of rice blast infection. This outcome underscores the negative regulatory role of *MOIRAs* in rice blast resistance, suggesting that these lncRNAs act as important modulators of the plant’s defense mechanisms.

### 3.4. Association of MOIRAs with Yield Traits in Rice

The delicate balance between disease resistance and yield has been a focal point in crop genetic breeding research, as resistance and yield traits typically present a trade-off. As our above results unveiled the negative regulatory roles of the three lncRNAs, *MOIRA1*, *MOIRA2*, and *MOIRA3*, in rice blast disease resistance, we were also keen to investigate their potential impacts on rice yield-related traits.

**Figure 5:**
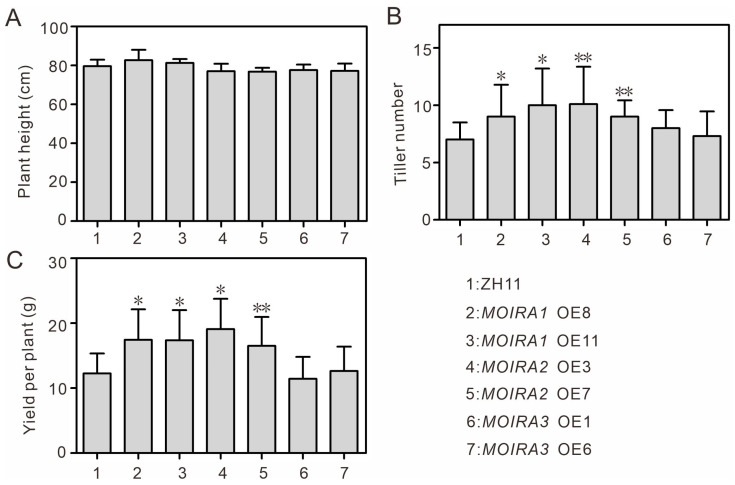
Phenotypic analyses of ZH11 and *MOIRA*-overexpressing plants under normal growth conditions. Statistical data for plant height (**A**), tiller number (**B**), and yield per plant (**C**) of ZH11 and *MOIRA*-overexpressing plants. Values are expressed as the means ± SD of three biological replicates (16 plants for each replicate). The asterisks indicate significant differences compared with the ZH11 plants (Dunnett’s test, ** *p* < 0.01 and * *p* < 0.05).

In pursuit of this, we closely examined yield-related traits in the *MOIRA*-overexpressing plants. Remarkably, overexpressing *MOIRA1* or *MOIRA2* significantly increased the tiller number in rice, consequently leading to a substantial enhancement in single-plant yield (Figure 5). Additionally, we examined other yield-related traits, such as plant height, grain length, and grain width, but did not observe any substantial variations (Figure 5 and Figure S1). This intriguing finding suggests that these lncRNAs may influence not only the plant’s response to pathogens, but also its overall productivity. The precise mechanisms underlying this association warrant further investigation, but hold promise for the development of rice cultivars with improved yield potential. This discovery highlights the potential for breaking the traditional trade-off between disease resistance and crop productivity, contributing to advancements in crop genetic breeding.

## 4. Discussion

The roles of non-coding RNAs, such as lncRNAs, in the fine-tuning and regulation of gene expression, have profound implications for crop genetic improvement. The conventional approach to crop breeding has largely focused on protein-coding genes as the primary targets for enhancement. However, the subtle and intricate regulatory roles of non-coding RNAs are now gaining recognition as pivotal components of crop development and adaptation to environmental challenges. The discovery of three lncRNAs, collectively known as *MOIRAs*, responsive to *M. oryzae* infection in rice, represents a significant breakthrough in the understanding of plant–pathogen interactions. While previous studies have often focused on the correlation between rice lncRNAs and their responses to rice blast disease, this research marks the first to report the biological functionality of lncRNAs in rice blast resistance. The overexpression of these lncRNAs resulted in a substantial increase in rice susceptibility to the disease. However, the specific molecular mechanisms underpinning this effect warrant further investigation.

Our study on *MOIRAs* exemplifies the importance of non-coding RNAs in orchestrating the complex interplay of molecular processes that determine disease resistance and yield traits in crops. The ability of these lncRNAs to act as molecular switches, shifting the balance between resistance and yield, underscores their significance in crop genetic improvement. The fine-tuning effect of non-coding RNAs presents a promising avenue for breeding crops that are not only resistant to diseases, but also productive [25,26,27]. This revelation suggests that, in addition to traditional breeding methods, manipulating the expression, sequence, or structural aspects of non-coding RNAs like *MOIRAs* may offer a new strategy through which to uncouple the trade-off between disease resistance and growth. Such an approach holds great potential for developing crop varieties that simultaneously exhibit high yields and robust disease resistance, thus addressing a critical challenge in crop genetic improvement.

The delicate equilibrium between disease resistance and yield has long been a focal point of crop genetic research [28,29,30,31,32]. Plant diseases not only pose a substantial threat to crop yield, but also undermine food security, making the development of novel strategies for disease control imperative. While previous research has identified several key disease resistance genes, their application has been hampered by the inherent conflict between disease resistance and growth and development. Our study offers a promising resolution to this age-old conflict. *MOIRA1* and *MOIRA2*, in addition to their roles in regulating disease susceptibility, have been found to significantly increase rice tillering and, subsequently, single-plant yield. This dual functionality of *MOIRAs* implies that they serve as crucial nodes in coordinating resistance and productivity. In the future, it may be possible to manipulate *MOIRA1* and *MOIRA2* through precise regulatory approaches, such as altering their temporal and spatial expression or modifying their sequences and structures. Such interventions hold the potential to break the coupling between yield and disease resistance, providing a novel strategy for developing crop materials that exhibit both high productivity and resilience to diseases.

In addition, our study has shed light on the adverse regulatory role of *MOIRAs* in rice blast resistance. While their overexpression enhances susceptibility, it raises the question of whether the deletion of these lncRNAs could confer rice with a corresponding resistance. The multifaceted participation of lncRNAs in disease resistance represents a promising avenue for gaining profound insights into plant defense mechanisms. Future research endeavors should delve deeper into the intricate molecular pathways through which *MOIRAs* exert their influence on resistance and explore the potential for manipulating these lncRNAs to enhance disease resistance in rice. This exploration opens new horizons for advancing our understanding and application of lncRNA-based strategies in crop protection.

The integration of *MOIRAs’* molecular insights with state-of-the-art breeding techniques represents a promising frontier in crop genetic improvement. This synergy between our understanding of *MOIRAs* and modern biotechnological tools, particularly CRISPR-Cas9 gene editing and marker-assisted selection, holds the potential to revolutionize rice breeding and beyond. However, it is important to note that, as of now, the full extent of *MOIRAs’* functionality and underlying mechanisms remain the subjects of ongoing research. Therefore, while these approaches hold exciting promise, the specific applications of *MOIRAs* in future breeding programs are contingent on a more comprehensive understanding of their roles and interactions. It is worth exploring the concept that *MOIRAs* may function as “tolerance non-coding sequences”, contributing not only to disease resistance, but also potentially playing a role in plant–pathogen coevolution. The dual functionality of *MOIRAs*, influencing both resistance and yield traits, aligns with the intricate balance between plants and pathogens in their continuous coevolution. By integrating CRISPR-Cas9 gene editing and marker-assisted selection with the insights gained from *MOIRA* research, breeders can navigate the complex dynamics between resistance and yield, offering a holistic approach to enhancing crop productivity and resilience.

In conclusion, this study not only deepens our understanding of non-coding RNAs’ roles in plant defense mechanisms, but also offers a potential solution to the long-standing challenge of balancing crop resistance and yield. By fine-tuning the expression and function of lncRNAs like *MOIRA1* and *MOIRA2*, we may unlock the potential for developing crops that are both highly productive and resilient in the face of diseases, contributing to global food security and sustainable agriculture.

## Data Availability

The data presented in this study are available on request from the corresponding author Qing Liu.

## Supplementary Materials

The following supporting information can be downloaded at: https://www.mdpi.com/article/10.3390/genes15010082/s1, Figure S1. Grain length and width of ZH11 and lncRNA-overexpressing plants under normal growth conditions; Table S1. Primers used for vector construction and gene expression analysis in this study; Table S2. DNA loci and lncRNA sequences of *MOIRAs*.

## References

[1] Burdon J.J., Thrall P.H. (2009). Coevolution of plants and their pathogens in natural habitats. Science.

[2] Xue J., Lu Z., Liu W., Wang S., Lu D., Wang X., He X. (2021). The genetic arms race between plant and Xanthomonas: Lessons learned from TALE biology. Sci. China Life Sci..

[3] Asibi A.E., Chai Q., Coulter J.A. (2019). Rice blast: A disease with implications for global food security. Agronomy.

[4] Fernandez J. (2023). The Phantom Menace: Latest findings on effector biology in the rice blast fungus. aBIOTECH.

[5] Nejat N., Mantri N. (2018). Emerging roles of long non-coding RNAs in plant response to biotic and abiotic stresses. Crit. Rev. Biotechnol..

[6] Zhang H., Guo H., Hu W., Ji W. (2020). The emerging role of long non-coding RNAs in plant defense against fungal stress. Int. J. Mol. Sci..

[7] Yang Y., Liu T., Shen D., Wang J., Ling X., Hu Z., Chen T., Hu J., Huang J., Yu W. (2019). Tomato yellow leaf curl virus intergenic siRNAs target a host long noncoding RNA to modulate disease symptoms. PLoS Pathog..

[8] Song L., Fang Y., Chen L., Wang J., Chen X. (2021). Role of non-coding RNAs in plant immunity. Plant Commun..

[9] Feng Y.-Z., Zhu Q.-F., Xue J., Chen P., Yu Y. (2023). Shining in the dark: The big world of small peptides in plants. aBIOTECH.

[10] He H., Zhou Y.-F., Yang Y.-W., Zhang Z., Lei M.-Q., Feng Y.-Z., Zhang Y.-C., Chen Y.-Q., Lian J.-P., Yu Y. (2021). Genome-Wide Analysis Identified a Set of Conserved lncRNAs Associated with Domestication-Related Traits in Rice. Int. J. Mol. Sci..

[11] Yu Y., Zhang Y., Chen X., Chen Y. (2019). Plant noncoding RNAs: Hidden players in development and stress responses. Annu. Rev. Cell Dev. Biol..

[12] Wu L., Liu S., Qi H., Cai H., Xu M. (2020). Research progress on plant long non-coding RNA. Plants.

[13] Wierzbicki A.T., Blevins T., Swiezewski S. (2021). Long noncoding RNAs in plants. Annu. Rev. Plant Biol..

[14] Liu N., Xu Y., Li Q., Cao Y., Yang D., Liu S., Wang X., Mi Y., Liu Y., Ding C. (2022). A lncRNA fine-tunes salicylic acid biosynthesis to balance plant immunity and growth. Cell Host Microbe.

[15] Jiang N., Cui J., Hou X., Yang G., Xiao Y., Han L., Meng J., Luan Y. (2020). Sl-lncRNA15492 interacts with Sl-miR482a and affects Solanum lycopersicum immunity against Phytophthora infestans. Plant J..

[16] Jiang N., Cui J., Shi Y., Yang G., Zhou X., Hou X., Meng J., Luan Y. (2019). Tomato lncRNA23468 functions as a competing endogenous RNA to modulate NBS-LRR genes by decoying miR482b in the tomato-Phytophthora infestans interaction. Hortic. Res..

[17] Zhou C., Zhu J., Qian N., Guo J., Yan C. (2021). Bacillus subtilis SL18r Induces Tomato Resistance Against Botrytis cinerea, Involving Activation of Long Non-coding RNA, MSTRG18363, to Decoy miR1918. Front Plant Sci.

[18] Zhang L., Wang M., Li N., Wang H., Qiu P., Pei L., Xu Z., Wang T., Gao E., Liu J. (2018). Long noncoding RNAs involve in resistance to Verticillium dahliae, a fungal disease in cotton. Plant Biotechnol. J..

[19] Wang L.-L., Jin J.-J., Li L.-H., Qu S.-H. (2020). Long Non-coding RNAs Responsive to Blast Fungus Infection in Rice. Rice.

[20] Jain P., Sharma V., Dubey H., Singh P.K., Kapoor R., Kumari M., Singh J., Pawar D.V., Bisht D., Solanke A.U. (2017). Identification of long non-coding RNA in rice lines resistant to Rice blast pathogen Maganaporthe oryzae. Bioinformation.

[21] Choi G., Jeon J., Lee H., Zhou S., Lee Y.-H. (2022). Genome-wide profiling of long non-coding RNA of the rice blast fungus Magnaporthe oryzae during infection. BMC Genom..

[22] Liang M., Ye H., Shen Q., Jiang X., Cui G., Gu W., Zhang L.H., Naqvi N.I., Deng Y.Z. (2021). Tangeretin inhibits fungal ferroptosis to suppress rice blast. J. Integr. Plant Biol..

[23] Liu Q., Yang T., Yu T., Zhang S., Mao X., Zhao J., Wang X., Dong J., Liu B. (2017). Integrating small RNA sequencing with QTL mapping for identification of miRNAs and their target genes associated with heat tolerance at the flowering stage in rice. Front. Plant Sci..

[24] Yu Y., Zhou Y.-F., Feng Y.-Z., He H., Lian J.-P., Yang Y.-W., Lei M.-Q., Zhang Y.-C., Chen Y.-Q. (2020). Transcriptional landscape of pathogen-responsive lncRNAs in rice unveils the role of ALEX1 in jasmonate pathway and disease resistance. Plant Biotechnol. J..

[25] Datta R., Paul S. (2019). Long non-coding RNAs: Fine-tuning the developmental responses in plants. J. Biosci..

[26] Ariel F., Romero-Barrios N., Jégu T., Benhamed M., Crespi M. (2015). Battles and hijacks: Noncoding transcription in plants. Trends Plant Sci..

[27] Waititu J.K., Zhang C., Liu J., Wang H. (2020). Plant non-coding RNAs: Origin, biogenesis, mode of action and their roles in abiotic stress. Int. J. Mol. Sci..

[28] Ning Y., Liu W., Wang G.-L. (2017). Balancing immunity and yield in crop plants. Trends Plant Sci..

[29] Chandran V., Wang H., Gao F., Cao X.-L., Chen Y.-P., Li G.-B., Zhu Y., Yang X.-M., Zhang L.-L., Zhao Z.-X. (2019). miR396-OsGRF s module balances growth and rice blast disease-resistance. Front. Plant Sci..

[30] Li Y., Zheng Y.-P., Zhou X.-H., Yang X.-M., He X.-R., Feng Q., Zhu Y., Li G.-B., Wang H., Zhao J.-H. (2021). Rice miR1432 fine-tunes the balance of yield and blast disease resistance via different modules. Rice.

[31] Xiao N., Pan C., Li Y., Wu Y., Cai Y., Lu Y., Wang R., Yu L., Shi W., Kang H. (2021). Genomic insight into balancing high yield, good quality, and blast resistance of japonica rice. Genome Biol..

[32] Zhu Q., Feng Y., Xue J., Chen P., Zhang A., Yu Y. (2023). Advances in Receptor-like Protein Kinases in Balancing Plant Growth and Stress Responses. Plants.

