# Supplementary material for: Unveiling the Roles of LncRNA MOIRAs in Rice Blast Disease Resistance
Source: Genes (Basel). 2024 Jan 9;15(1):82. doi: 10.3390/genes15010082 (PMC10815219; doi:10.3390/genes15010082)
Supplement: Supplementary file 1 [file genes-15-00082-s001.zip › genes-2732395-supplementary.pdf]

Table S1. Primers used for vector construction and gene expression analysis in this study.

Gene	Forward primer (5'-3')	Reverse primer (5'-3')
XLOC_083948-OE	GGTGTTACTTCTGCAGCCTGACAGCGCAGCATCTTGT	CGCCGGTACCCTGCAGTCCTCCTCCTCCTCCTCCC
XLOC_419751-OE	GGTGTTACTTCTGCAGCTATCGTCCATATGGATTGTACCG	CGCCGGTACCCTGCAGTTGCTTTGTAGGTGGAGATCAAGA
XLOC_428992-OE	GCTTACTAGTACGCGTGGAGATTGGCCGTCATCGTGAA	AGGATCCATAACGCGTTACGGTGATCCGGCGGCAA
XLOC_083948-qRT-PCR	TCTTCTTGCAAGCACCCACA	CCACGAGTGTTACCACATCAC
XLOC_419751-qRT-PCR	CCCCTGCTTCTTTCAGGTGT	GATGAGAGCTTTGGTGGCCT
XLOC_428992-qRT-PCR	CCATCTCCTTCGCCGTTCAT	GATCCGGCGGCAACACAT
OsPR1a	ACGTACGTATGCTGGTGAGA	TGCATGTAACCACGAAGGACA
OsPR1a-2	CCCTTTTTAATTAGCAATTAGAGAGCT	CGAGCACTGCAGCTACTAGCAAGCTG
OsPR1b	TATCCAAGCTGGCCATTGCT	TTCTCTGGCTGGCGTAGTTC
OsPR5	CCAAGTCGTCTTCTGCCCAT	CAATTGCACACGTGGTCGAG
EF1α	TTTCACTCTTGGTGTGAAGCAGAT	GACTTCCTTCACGATTTCATCGTAA

Table S2. DNA loci and lncRNA sequences of *MOIRAs*

	LncRNA ID	Length	Chromosome	Start	End	LncRNA sequence
MOIRA1	XLOC_083948	327	Chr10	19933213	19933791	CCUGACAGCGCAGCAUCUUGUUUGUUUUAUCUCCCACAUCAUCUCCCUCAUC<br>AUCAUCAUCAUCUCCUCUUCUUGCAAGCACCCACAACAACACAAACUCUCUU<br>UCUUCAUCUUUCUCUCUCCUCCUCCUCUCUUUGUUGUACUCUACUCAACAUU<br>UCUCAACUUUCACUUCUUCUUCUUCUUCUUCCUUUCUCUCUUCACAGCUAUC<br>AUAUUCUCCUCCUGCUUGCUGCUGCUGCUGCUGGCUGUUUCUCUCUCAUCCA<br>UCCACCCUAUUAUUUAUUCAAGGCUAAGUGAUGUGGUAACACUCGUGGGGG<br>AGGAGGAGGAGGAGGA
MOIRA2	XLOC_419751	557	Chr8	24315675	24316231	CUAUCGUCCAUAUGGAUUGUACCGGCCUGAACCACCAGCCAAAUUUCCCCUG<br>CUUCUUUCAGGUGUGCUACCAGAUGCAUAACCACCUAGUGCAGGAUCAUAU<br>GCUGCGCCGCCAUACAUAGCGCCACCAAAGCUAGAACUACUGAGGCCACCAA<br>AGCUCUCAUCACCACCACGCCUGUAUCCACUUAAGUUCCCACCAGCGUAAGG<br>UCCAUCAAAACCUCCUCCAUACGGCCCAACACGACUAGCAUAACCAAAAGAA<br>GGCAUGCUUCCAGAUCCUCCUAAUCCUGGAUAGUAACGGCCAUACUGAACA<br>CCAUAUUCACCUAUCCCACCAUAACCACUGAGACUACCACCACCAUAAACUC<br>CAUGGUUCCUAUAAGGCCCAAAUCCACCACUGUUGUAGUUGCUAUAAGCAU<br>UGGCCAGUCCACCAUAGUCAUUGCCAGAAGAAUGACCCCUAGAAUCCCUACC<br>AUAUGCUGAUCUAGAGGCACUACCAUGUGAUGAUGGUGGUGGAUUUGAGGA<br>UUUCUUUGGUUCUGCUUUCUUGAUCUCCACCUACAAAGCAA
MOIRA3	XLOC_428992	255	Chr8	10531583	10531985	GGAGAUUGGCCGUCAUCGUGAACCGGAGCGCCGCCGUCGCCGCCGUGCCUCC<br>AGCCGCGCCGUUCGUCGUCGUCCGUCGUCACCUUCGGUCGCCGUCGUCUCCA<br>UUUGAUUCGCAGCUGCGAGGAGAAGCCCGUCCGCCAUCUGUCAUCCGCCGA<br>AGAUCGCCGGAAAGUCGUCGCCGGCCAUCUCCUUCGCCGUUCAUCGCUGCGU<br>AAGUAUUGCAUCGUCGUCGUCUCCAUGUGUUGCCGCCGGAUCACCGUA

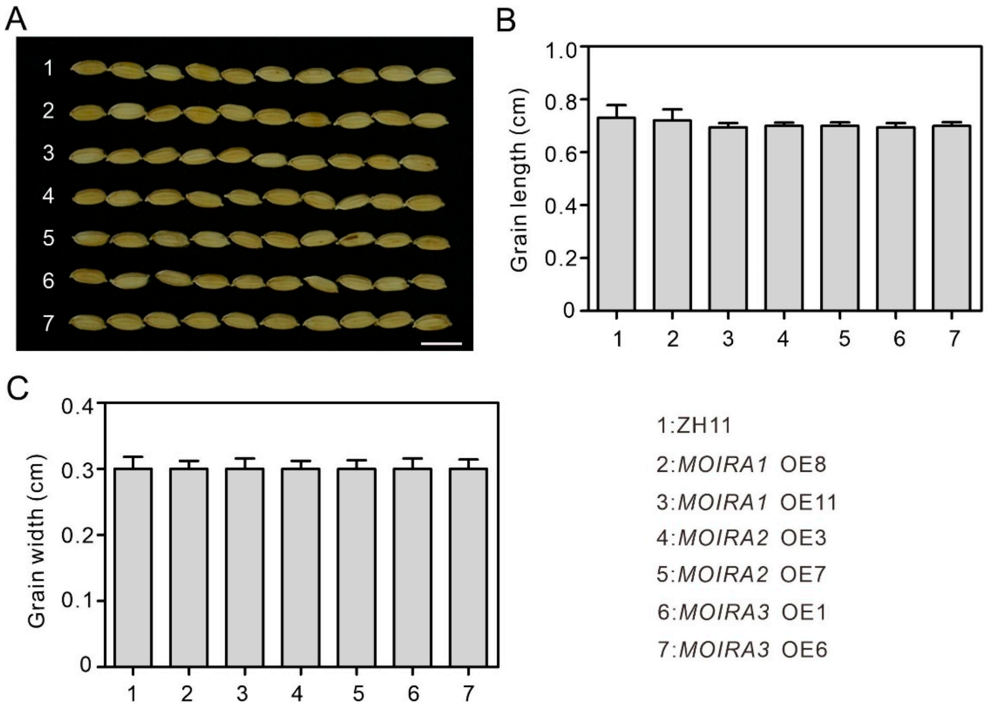

Figure S1. Grain length and width of ZH11 and lncRNA-overexpressing plants under normal growth conditions. A. Grain morphology of ZH11 and lncRNA overexpressing plants. Scale bar = 1.0 cm. B. Grain length of ZH11 and lncRNA overexpressing plants. C. Grain width of ZH11 and lncRNA overexpressing plants. Values are means ± SD of three biological replicates (30 grains for each replicate) and asterisks indicate significant differences between ZH11 and overexpressing plants (Dunnett's test, ***P* < 0.01 and **P* < 0.05).
